# Genome signature analysis of thermal virus metagenomes reveals Archaea and thermophilic signatures

**DOI:** 10.1186/1471-2164-9-420

**Published:** 2008-09-17

**Authors:** David T Pride, Thomas Schoenfeld

**Affiliations:** 1 Department of Medicine, Division of Infectious Diseases and Geographic Medicine, Stanford University School of Medicine, Stanford, CA, USA; 2 Lucigen, Middleton, WI, USA

## Abstract

**Background:**

Metagenomic analysis provides a rich source of biological information for otherwise intractable viral communities. However, study of viral metagenomes has been hampered by its nearly complete reliance on BLAST algorithms for identification of DNA sequences. We sought to develop algorithms for examination of viral metagenomes to identify the origin of sequences independent of BLAST algorithms. We chose viral metagenomes obtained from two hot springs, Bear Paw and Octopus, in Yellowstone National Park, as they represent simple microbial populations where comparatively large contigs were obtained. Thermal spring metagenomes have high proportions of sequences without significant Genbank homology, which has hampered identification of viruses and their linkage with hosts. To analyze each metagenome, we developed a method to classify DNA fragments using genome signature-based phylogenetic classification (GSPC), where metagenomic fragments are compared to a database of oligonucleotide signatures for all previously sequenced Bacteria, Archaea, and viruses.

**Results:**

From both Bear Paw and Octopus hot springs, each assembled contig had more similarity to other metagenome contigs than to any sequenced microbial genome based on GSPC analysis, suggesting a genome signature common to each of these extreme environments. While viral metagenomes from Bear Paw and Octopus share some similarity, the genome signatures from each locale are largely unique. GSPC using a microbial database predicts most of the Octopus metagenome has archaeal signatures, while bacterial signatures predominate in Bear Paw; a finding consistent with those of Genbank BLAST. When using a viral database, the majority of the Octopus metagenome is predicted to belong to archaeal virus Families *Globuloviridae *and *Fuselloviridae*, while none of the Bear Paw metagenome is predicted to belong to archaeal viruses. As expected, when microbial and viral databases are combined, each of the Octopus and Bear Paw metagenomic contigs are predicted to belong to viruses rather than to any Bacteria or Archaea, consistent with the apparent viral origin of both metagenomes.

**Conclusion:**

That BLAST searches identify no significant homologs for most metagenome contigs, while GSPC suggests their origin as archaeal viruses or bacteriophages, indicates GSPC provides a complementary approach in viral metagenomic analysis.

## Background

The study of metagenomes has provided important insights into physiological processes and into the diversity of microbial and viral communities in different environments [1,2]. Metagenomic analysis is based on high-throughput DNA sequencing of clone libraries of mass-isolated cells or viral particles from different ecological environments, and is strictly defined as the study of those organisms that inhabit a given biological niche. Such community analysis has contributed to an improved understanding of microbial community structure, and can provide a broader perspective on microbial community composition and function than analysis of 16s rDNA.

Over the past decade, it has become increasingly clear that viruses are a significant component of every ecological niche in which cellular life exists. Abundances ranging from 10^4 ^to 10^8 ^virus-like-particles per milliliter have been detected in virtually every aquatic environment studied [3], although abundances in hot springs are generally at the lower end of this range [4]. Estimates of viral diversity suggest that several thousand different viral types exist in a given pool, probably having a profound impact on population structure and genomic content of host populations [5-8].

Studies of viral diversity have been hampered by the absence of universal signature sequences (*e.g. *16S rDNA). Metagenomic analysis has provided much of the population-level insight into diversity and distribution of viruses in the environment [9]. The few studies addressing bacteriophage and archaeal viral assemblages have led to deeper understandings of the diversity present in these communities and may aid in the determination of how the presence of certain viruses may shape microbial communities [7,10]; however, these studies also have highlighted the need for improved approaches in the analysis of viral metagenomes. In each of the studied viral metagenomes, a large proportion of sequences had no significant homologs identified in Genbank non-redundant database [9,11-13]. Furthermore, in a recent viral metagenome survey in thermal environments, half of the sequences had no BLASTx homolog in the Genbank nr database [12], similar to results found in marine and estuarine environments [9,11], presumably due to the relative dearth of annotated thermophilic viral sequences in Genbank. While all of the unidentified sequences in thermal virus metagenomes presumably represent bacteriophage or archaeal viruses, neither the host nor types of virus can be ascertained [4,12]. Since, to date, BLAST alignments [14] have been the predominant means of associating a viral metagenomic sequence with a likely host, the lack of significant homology between most of viral metagenomic sequences and sequences in Genbank has impeded our understanding of host-virus relationships.

Genome signature analysis of DNA sequences measures biases in DNA oligonucleotide composition rather than sequence similarity, and is studied in an alignment-independent manner [15-18]. For each genome or portion of genome with detectable differences, the genome signature for each sequence analyzed will be unique [15,19]. Previous data has demonstrated that after their divergence, microbes retain patterns of genome signature reflective of their recent common ancestry similar to that of 16s rDNA [15]. Utilizing this quality of the genome signature, the technique now has been adapted to predict the ancestry of eukaryal, archaeal, and bacterial metagenomic sequences [20].

The classification of viruses has traditionally been based on morphological characteristics [21,22]. This classification system is widely used for cultivated viruses, which significantly biases our view of diversity [23]. Attempts have been made to correlate sequences and morphologies [24], but these have proven less useful in extreme thermal environments. The absence of a universal signature gene has hampered classification of viral genomes based on genomic sequences Recent studies of bacteriophages have identified conserved patterns of oligonucleotides used as genome signatures unique to each genome analyzed that appear to be co-evolving with their hosts [25]. In contrast, these patterns are shared for groups of eukaryotic viruses in a manner largely independent of their host [25].

Terrestrial thermal aquifers are vast ecosystems with abundances of microbes and viruses approaching those of the ocean [4,12]. At temperatures > 74°Celsius, the hot springs in this study are significantly above the temperature limit for eukaryotic life, generally accepted to be around 62°C, and therefore, harbor communities strictly composed of Bacteria, Archaea, and their respective viruses [26]. While comprehensive studies of viral communities in these extreme environments are just beginning, culture-based studies have indicated the presence of bacteriophages of the bacterial Genus *Thermus *[27,28], as well as archaeal viruses of the archaeal Genera *Sulfolobus*, *Pyrobaculum*, *Acidianus*, and *Thermoproteus *[29].

We sought to develop new methods based on genome signature to apply to analysis of viral communities from two separate thermal pools, Bear Paw and Octopus Springs, in Yellowstone National Park. Our goals were to: 1) develop the technique of genome signature-based phylogenetic classification (GSPC) to accurately predict the presumed host/virus relationships of known bacteriophages, 2) analyze the differences between viral metagenomes from Bear Paw and Octopus hot springs, 3) apply the GSPC technique to viral metagenomes to predict the microbial host of unknown members of the viral community, and 4) apply GSPC to classify the viruses present in Bear Paw and Octopus hot springs.

## Results

### GSPC for known bacteriophages based on a microbial database

To compare viral genome signatures with those of Bacteria and Archaea, we constructed a microbial database of oligonucleotide frequencies for all currently sequenced Bacteria and Archaea. The database contains frequencies of all dinucleotide, trinucleotide, tetranucleotide, pentanucleotide, and hexanucleotide combinations for each genome. To determine similarity between known bacteriophages and their potential hosts in the microbial database, Euclidean distances based on the sum of the differences for all oligonucleotide combinations were determined for each known bacteriophage and each database genome. The resulting distance matrix was subjected to neighbor-joining analysis, and phylogenies were used to classify the known bacteriophages. In cases where the known bacteriophages were positioned monophyletically, the bacteriophages were classified based on the Kingdom, Phylum, Class, Order, Family, and Genus of that monophyletic group. In cases where the known bacteriophages were positioned paraphyletically, the bacteriophages were classified based on the branches deep to that paraphyletic position.

**Figure 1:**
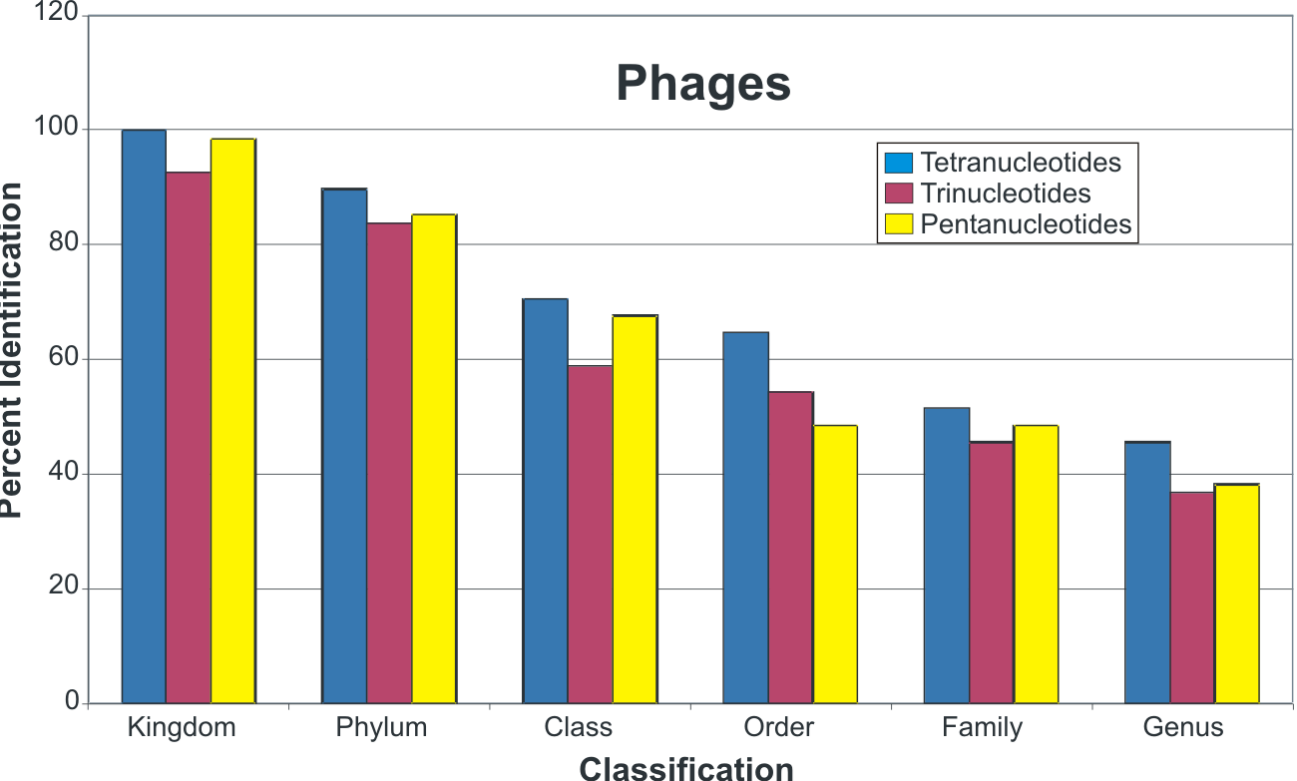
**Genome signature phylogenetic classification of known bacteriophages using a microbial database.** Each bacteriophage was subjected to genome signature classification as described in Materials and Methods. Bacteriophages were then classified according to their position on the genome signature phylogeny, and each position compared with that of the bacterial host in which they were originally isolated. The percentage of bacteriophages classified consistent with that of their bacterial hosts are represented. Blue represents bacteriophages classified by tetranucleotide GSPC, yellow represents bacteriophages classified by pentanucleotide GSPC, and red represents bacteriophages classified by trinucleotide GSPC.

Using a group of bacteriophages in which their bacterial host has been well-described (Additional file 1), GSPC was able to classify greater than 90% by Phylum, 70% by Class and Order, and 50% by Family and Genus (Figure 1). When analyzing by specific oligonucleotide sizes, tetranucleotide-based phylogeny classifies a higher percentage of bacteriophages than trinucleotides or pentanucleotides (Figure 1). Because of its increased sensitivity in classifying bacteriophages according to their bacterial hosts, tetranucleotide-based GSPC was used for all subsequent analyses.

### GSPC for hot spring metagenomes based on a microbial database

We analyzed two separate hot springs, Bear Paw and Octopus in Yellowstone National Park, to gain a deeper understanding of the viral populations native to each habitat. Each hot spring is located within 5 kilometers of the other, with Bear Paw characterized by a surface temperature of 74°Celsius, visible pigmented microbial growth at the surface, a pH near 8.0, and estimated phage abundance from 10^5 ^to 10^6 ^particles per milliliter. The Octopus hot spring is characterized by a pH near 8.0, a surface temperature of 93°Celsius, an estimated viral abundances from 10^5 ^to 10^6 ^particles per milliliter, and no visible growth at the surface [30]. Both thermophilic Bacteria and Archaea previously have been identified in Octopus hot spring, with *Thermocrinis *and *Aquificales *predominating among the sediment and filament Bacteria [31-33]. Due to the high temperatures, these hot springs are both devoid of eukaryotic life [26].

**Figure 2:**
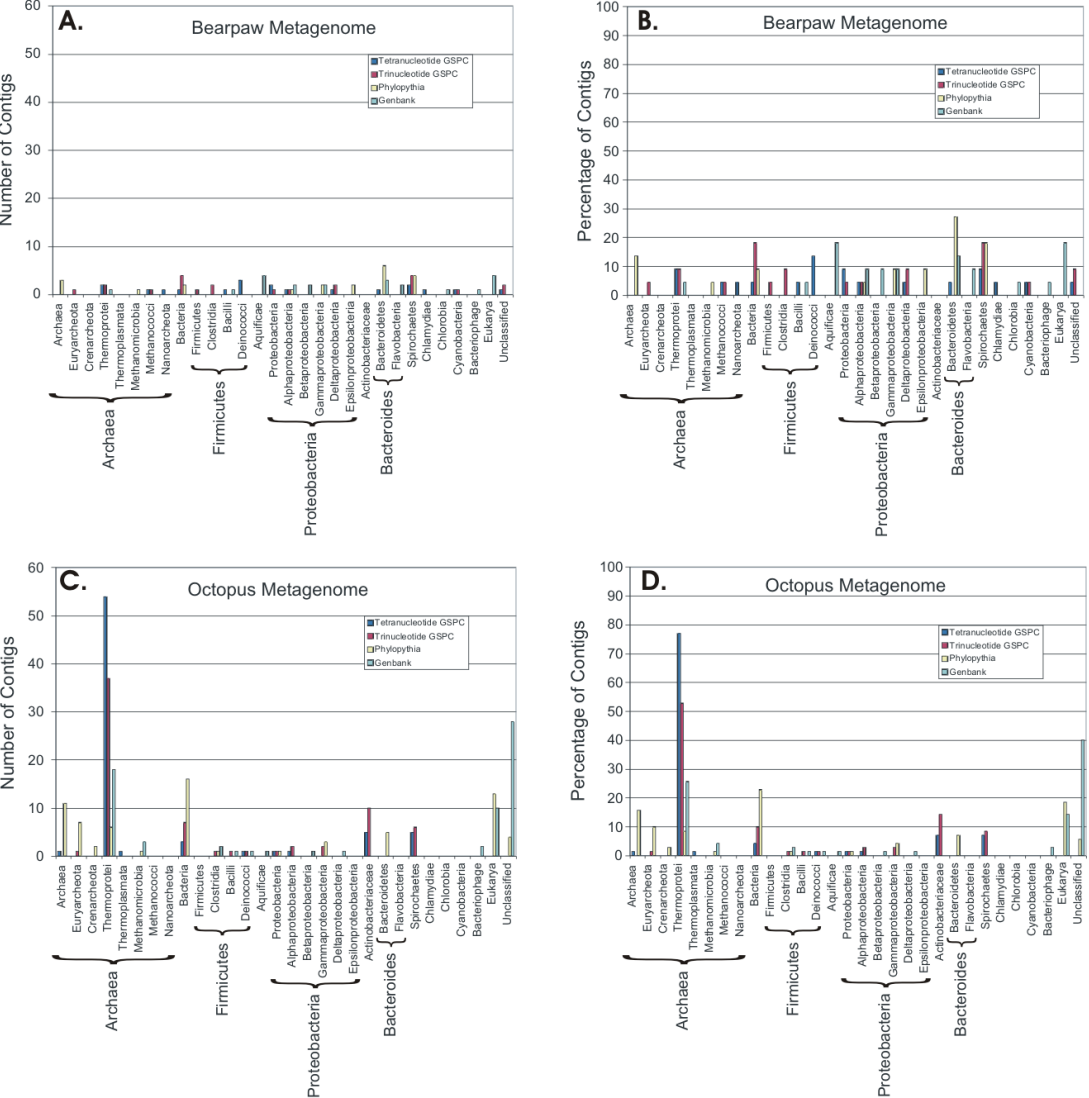
**Genome signature phylogenetic classification of contigs from Bear Paw and Octopus metagenomes using a microbial database.** Each contig was subjected to genome signature classification as described in Materials and Methods. The resulting number of contigs or percentage of the Octopus or Bear Paw contigs is presented by Class. Some methods could not classify certain metagenomic contigs beyond the level of Kingdom. Those contigs are presented by Kingdom. Panel A – number of Bear Paw contigs, Panel B – percentage of Bear Paw contigs, Panel C – number of Octopus contigs, and Panel D – percentage of Bear Paw contigs. Blue represents tetranucleotide GSPC, red represents trinucleotide GSPC, yellow represents Phylopythia, and cyan represents Genbank BLAST.

**Figure 3:**
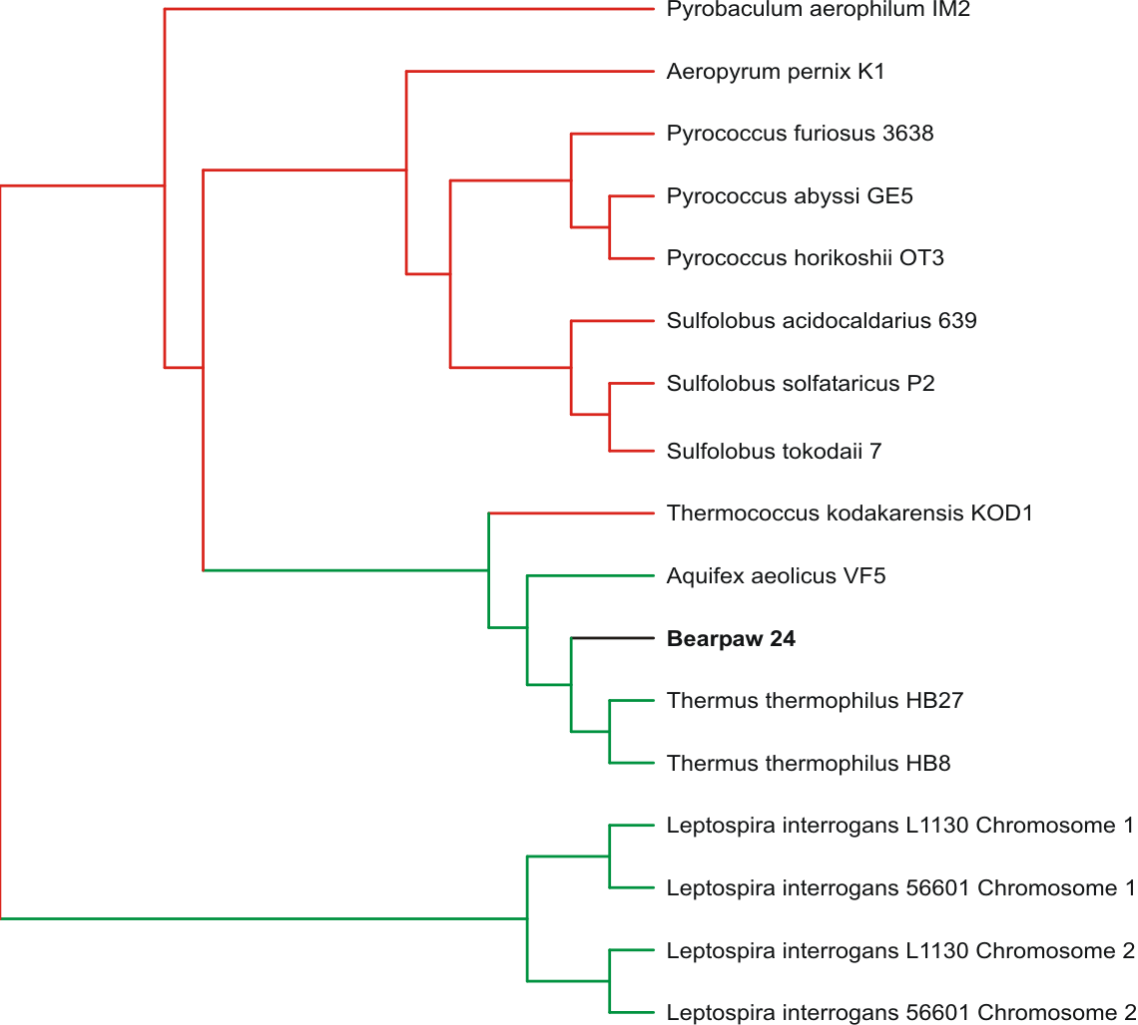
**Subtree of Bear Paw contig 24. **The metagenomic contig was subjected to oligonucleotide difference analysis at the tetranucleotide level, Euclidean distances computed, and compared by Neighbor-joining analysis with a microbial database. The resulting phylogeny contained 441 OTUs, and the portion of the phylogeny containing Bear Paw contig 24 is shown. Archaeal branches are shown in red, bacterial branches are shown in green, and the Bear Paw fragment is shown in black.

Using GSPC based on a microbial database, the majority of Bear Paw contigs are predicted to have bacterial hosts, while few are predicted to have archaeal hosts (Figure 2, Panels A and B). For Bear Paw contig 24, its phylogenetic position supports a classification with *Thermus thermophilus *(Figure 3), a thermophilic Bacterium highly characteristic of hot springs of similar temperature and water chemistry [34,35]. The closest homolog identified in Genbank for Bear Paw contig 24 belongs to another thermophilic Bacterium *Aquifex aeolicus *(Additional file 2). Both Genera *Aquifex *and *Thermus *are closely related based on genome signature-based phylogeny (Figure 3), despite their apparent divergence based on other criteria [36]. The fact that GSPC groups thermophilic Bacteria more closely with thermophilic Archaea than with mesophilic Bacteria is probably related to convergent evolution specific to thermophiles [37,38], but should not impair the predictive value of the method. GSPC based on a microbial database predicts other Bear Paw contigs belong to host bacterial Classes *Bacteroidetes*, *Alphaproteobacteria*, *Deltaproteobacteria*, and *Spirochaetes *(Figure 2, Panels A and B).

**Figure 4:**
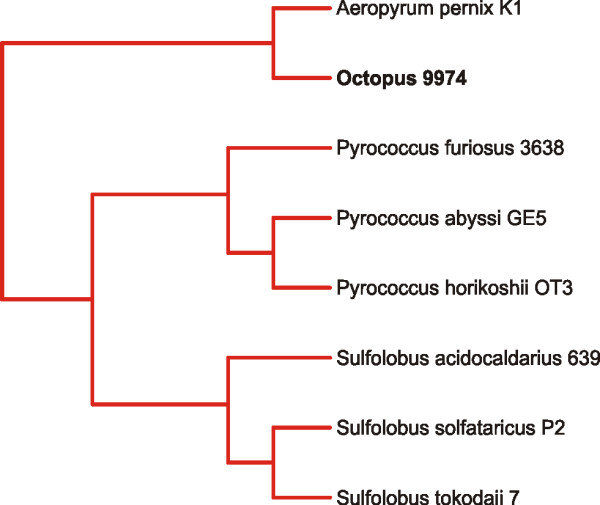
**Subtree of Octopus contig 9974.** The metagenomic contig was subjected to oligonucleotide difference analysis at the tetranucleotide level, Euclidean distances computed, and compared by Neighbor-joining analysis with a microbial database. The resulting phylogeny contained 441 OTUs, and the portion of the phylogeny containing Octopus contig 9974 is shown. Archaeal branches are shown in red, and the Octopus contig is shown in black.

When sequences from Octopus hot spring were analyzed by GSPC based on a microbial database, most were predicted to have archaeal hosts (Figure 2, Panels C and D). For Octopus contig 9974, its phylogenetic position supports a classification with *Aeropyrum pernix*, a hyper-thermophilic Archaeon belonging to the Class *Thermoprotei*, (Figure 4). Based on Genbank BLAST, the most closely related homolog of Octopus contig 9974 belongs to a virus isolated from the Archaeon *Sulfolobus islandicus *(Additional file 2), another hyper-thermophilic Archaeon belonging to the Class *Thermoprotei*. Most contigs from Octopus have substantial oligonucleotide similarity with the archaeal Genus *Pyrobaculum *(Additional file 2), which also belongs to the Class *Thermoprotei*. The abundance of contigs predicted to belong to the Genus *Pyrobaculum *suggests that *Pyrobaculum *viruses represent the most abundant viruses in Octopus hot spring. This is highly consistent with a previous metagenomic study of Octopus and Bear Paw hot springs in which homology to nearly the entire genome of *Pyrobaculum *spherical virus was detected [12,39]. Other Octopus contigs are predicted to belong to bacterial Classes *Actinobacteria*, *Spirochaetes*, and *Deinococci*, which includes the thermophilic Bacterium *Thermus thermophilus *(Figure 2, Panels C and D).

**Figure 5:**
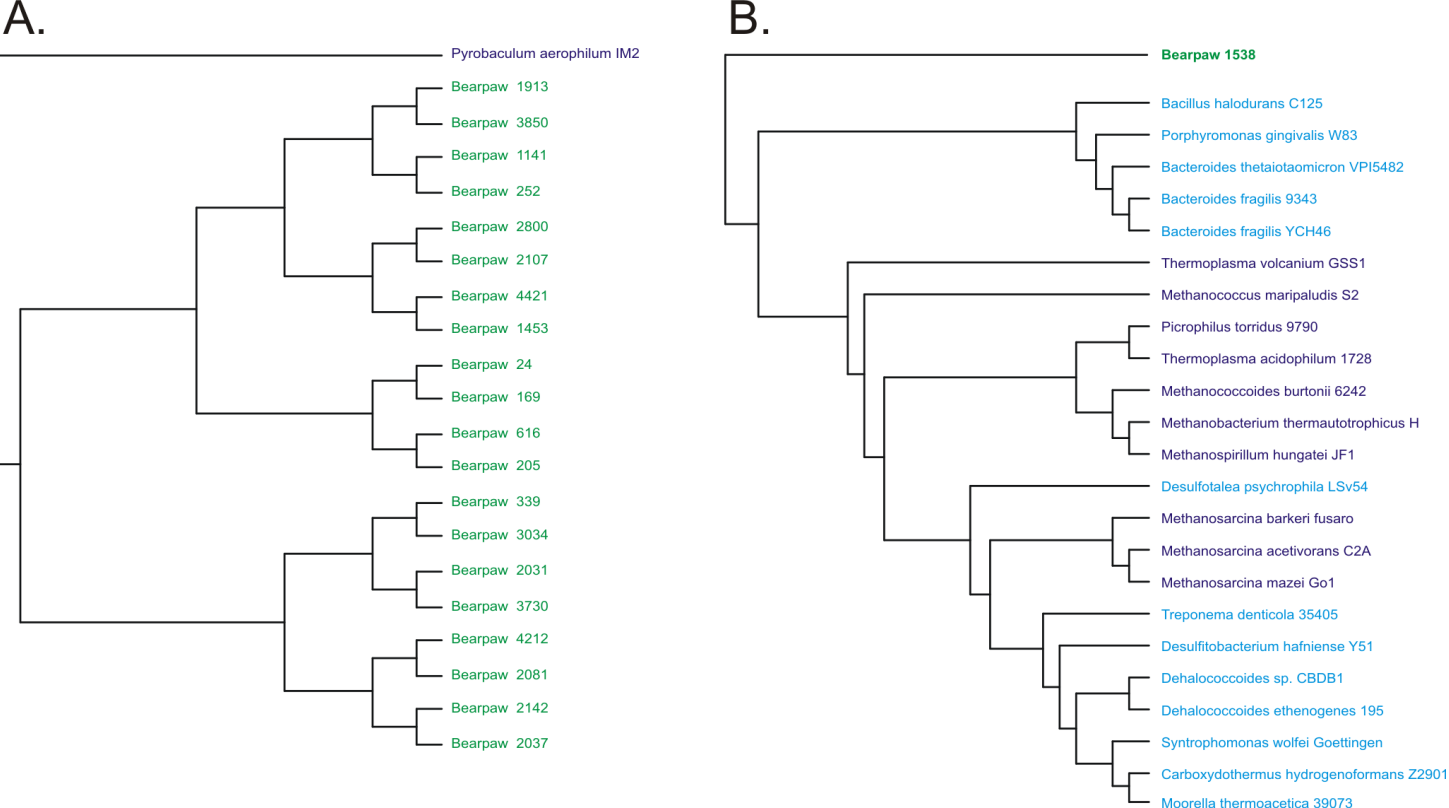
**Subtrees of Bear Paw metagenome contigs.** The metagenomic contigs were subjected to oligonucleotide difference analysis at the tetranucleotide level, Euclidean distances computed, and compared by Neighbor-joining analysis with a microbial database. The resulting phylogeny contained 462 OTUs, and the portions of the phylogeny containing the Bear Paw contigs are shown. Bear Paw contigs are shown in green, Archaea are shown in purple, and Bacteria are shown in blue. Panel A – all Bear Paw contigs except contigs 1538 and 697, Panel B – subtree containing the Bear Paw contig 1538.

**Figure 6:**
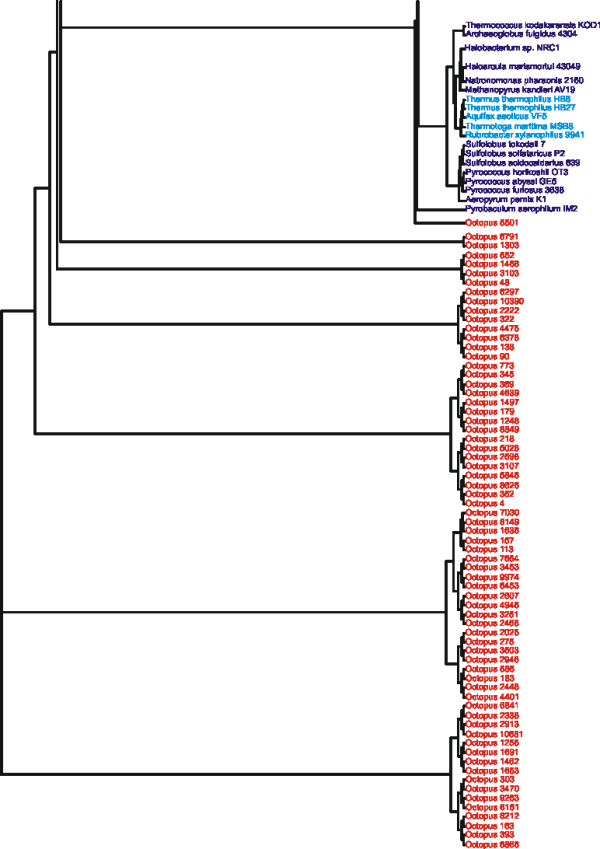
**Subtree of all Octopus metagenomic contigs.** The metagenomic contigs were subjected to oligonucleotide difference analysis at the tetranucleotide level, Euclidean distances computed, and compared by Neighbor-joining analysis with a microbial database. The resulting phylogeny contained 510 OTUs, and the portion of the phylogeny containing the metagenomic contigs is shown. Octopus contigs are shown in orange, Bacteria are shown in blue, and Archaea are shown in purple.

GSPC analysis classifies metagenomic contigs individually based on their similarity to other microbial genomes; however, classification also can be performed on the collective metagenomes. When the Bear Paw metagenomic contigs were analyzed collectively, the most recent common ancestor of each contig was found within the Bear Paw metagenome (Figure 5a), with the exception of contigs 697 and 1538 (Figure 5b). A similar finding is present for the Octopus metagenome, where the most recent common ancestor for each contig is represented in the Octopus metagenome rather than the microbial database (Figure 6). The collective Octopus metagenome is grouped paraphyletically to the archaeal Genus *Pyrobaculum *(Figure 6), consistent with the findings for each individual contig (Additional file 2).

### Collective analysis of Bear Paw and Octopus hot springs

**Figure 7:**
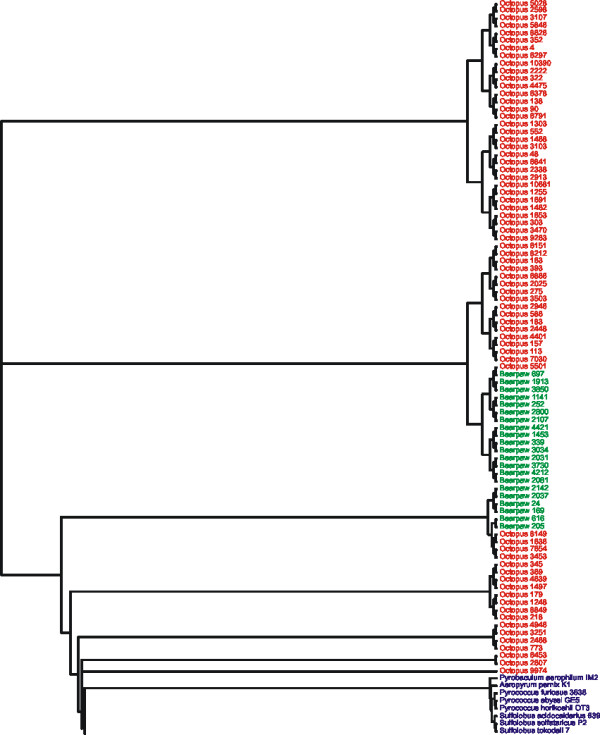
**Subtree of all Bear Paw and Octopus metagenomic contigs.** The metagenomic contigs were subjected to oligonucleotide difference analysis at the tetranucleotide level, Euclidean distances computed, and compared by Neighbor-joining analysis with a microbial database. The resulting phylogeny contained 532 OTUs, and the portion of the phylogeny containing the metagenomic contigs is shown. Bear Paw contigs are shown in green, Octopus contigs are shown in orange, and archaeal sequences are shown in purple.

When analyzed separately, contigs from Bear Paw and Octopus share a common genome signature with contigs from their respective metagenomes, suggesting that patterns of genome signature may be relatively specific to each environment. When metagenomes of Octopus and Bear Paw were analyzed together based on a microbial database, many of the Bear Paw contigs continue to demonstrate recent ancestry within the Bear Paw metagenome, with similar observations for the Octopus metagenome (Figure 7). Some overlap exists between groups of Octopus and Bear Paw contigs, suggesting the presence of shared microbial flora between the habitats (Figure 7), which is consistent with previous tBLASTx results [12].

### GSPC for known bacteriophages based on a viral database

Previous data indicates that when analyzing genome signature for diverse groups of viruses, they segregate largely according to their Family designation [25]. Furthermore, double stranded DNA viruses, including bacteriophages and archaeal viruses, cluster separately from other types of viruses such as single stranded DNA viruses and RNA viruses. Based on genome signature, bacteriophages typically segregate either according to their bacterial hosts or their Family designation (*e.g. Podoviridae*, *Myoviridae*, or *Siphoviridae*) [25].

**Figure 8:**
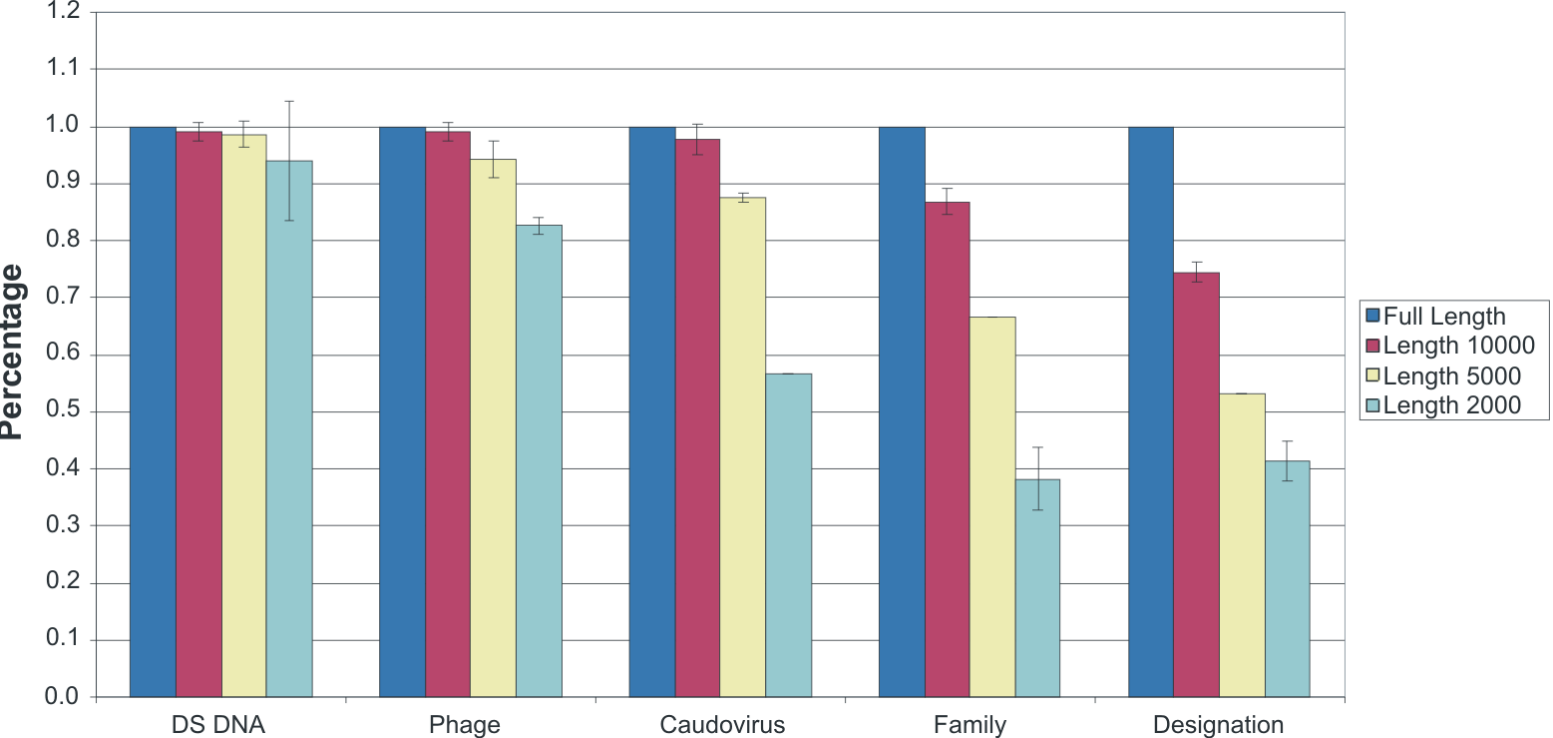
**Genome signature phylogenetic classification of known bacteriophages using a viral database.** Each bacteriophage fragment was subjected to genome signature classification as described in Materials and Methods. Each fragment was classified according to its position on the genome signature phylogeny, and each position compared with that of its classification based on morphological features. The percentage of bacteriophage fragments classified consistent with that of its morphological features is represented. Blue represents full length bacteriophages, red represents random bacteriophage fragments of 10,000 nucleotides, yellow represents random bacteriophage fragments of 5,000 nucleotides, and cyan represents random bacteriophage fragments of 2,000 nucleotides. Error bars represent standard error from a compilation of 5 separate experiments.

To classify metagenomic contigs according to their respective viral Families, we created a database of oligonucleotide signatures for all available sequenced viruses, and subjected a group of known bacteriophages (Additional file 1) to GSPC. Because the viral database contains all currently sequenced viruses, we selected random fragments of differing sizes from these viruses to evaluate their predicted ancestry. When full-length bacteriophages were evaluated, their predicted ancestry matches their ancestry based on morphological features (Figure 8). For bacteriophage fragments of 10,000 nucleotides, nearly 90% have predicted ancestry consistent with that of morphological features at the Family level. Most fragments of 5,000 nucleotides are predicted to be bacteriophages, with nearly 70% predicted according to Family. For fragments of 2,000 nucleotides, most are predicted to be bacteriophages; however, many are predicted to be bacteriophages outside of the *Caudoviridae *Family (Figure 8 and data not shown).

### GSPC for hot spring metagenomes based on a viral database

**Figure 9:**
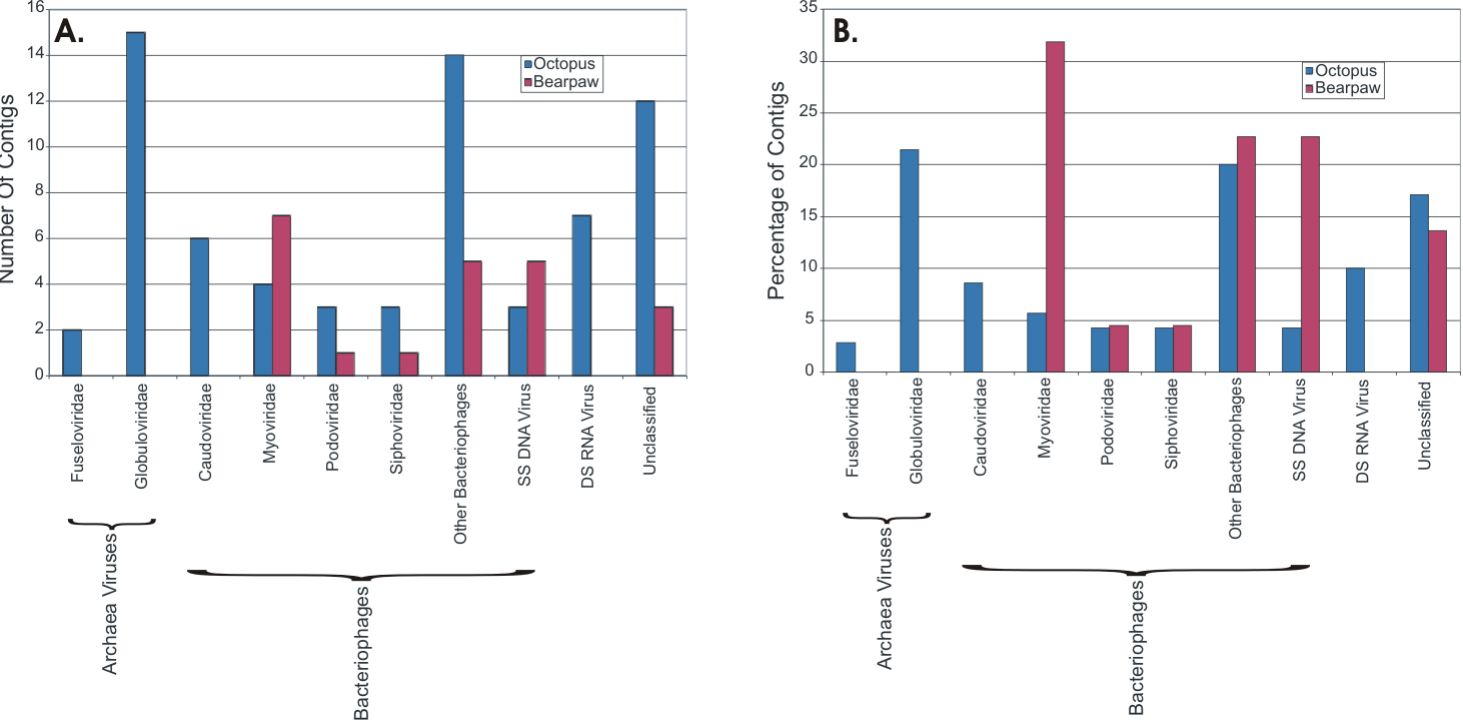
**Genome signature phylogenetic classification of contigs from Bear Paw and Octopus metagenomes using a viral database.** Each contig was subjected to genome signature classification as described in Materials and Methods. The resulting number of contigs or percentage of the Octopus or Bear Paw contigs is presented by Family. Panel A – number of contigs and Panel B – percentage of contigs. Blue represents Octopus contigs and red represents Bear Paw contigs.

**Figure 10:**
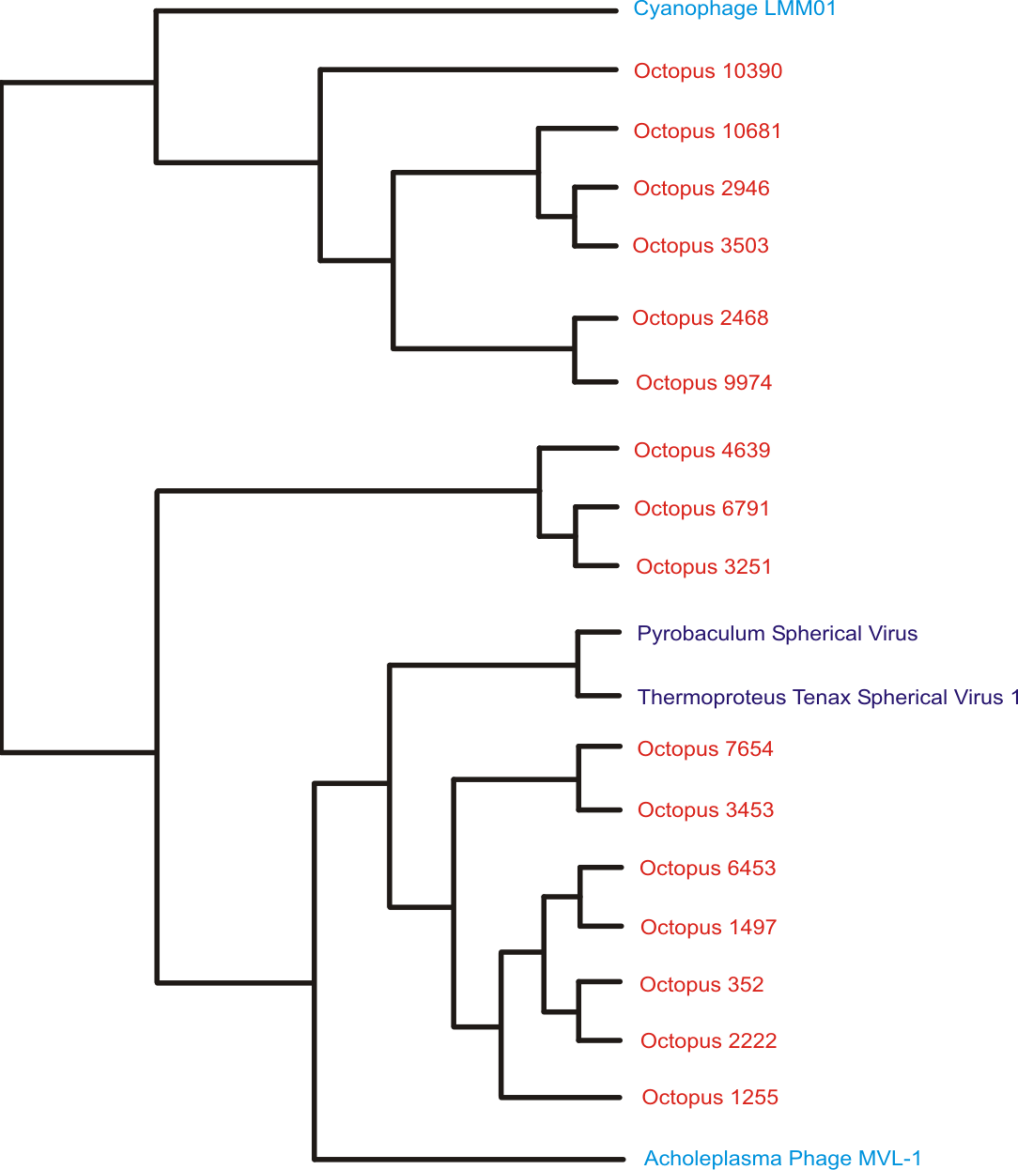
**Subtree of many Octopus metagenomic contigs.** The metagenomic contigs were subjected to oligonucleotide difference analysis at the tetranucleotide level, Euclidean distances computed, and compared by Neighbor-joining analysis with a viral database. The resulting phylogeny contained 3958 OTUs, and a portion of the phylogeny containing many Octopus contigs is shown. Octopus contigs are shown in orange, archaeal viruses are shown in purple, and bacteriophages are shown in blue.

We sought to predict the viral Families present in the Bear Paw and Octopus hot springs by subjecting contigs from both metagenomes to GSPC based on a viral database. As expected, the majority of the sequences from both Bear Paw and Octopus metagenomes are classified as double stranded DNA viruses (Figure 9), which is a result of selection due to the library construction method. Each of the common bacteriophage Families, including *Siphoviridae*, *Myoviridae*, and *Podoviridae *are predicted to be present in both metagenomes. The archaeal virus Family *Globuloviridae*, including *Thermoproteus *spherical virus and *Pyrobaculum *spherical virus, is substantially represented in Octopus hot spring (Figure 10), consistent with the predicted archaeal virus predominance when using a microbial database (Additional file 2). Another archaeal virus Family, *Fuselloviridae*, also is predicted to have members present in the Octopus metagenome (Figure 9). The majority of the Bear Paw metagenomic contigs are predicted to belong to the bacteriophage Family *Caudoviridae *(includes *Myoviridae*, *Podoviridae*, and *Siphoviridae*), with no individual contigs predicted to belong to archaeal viruses (Figure 9). The predicted bacteriophage predominance in Bear Paw and the predicted archaeal virus predominance in Octopus is consistent with the results of Genbank BLAST, and with well described correspondence between higher temperatures and higher predominance of Archaea [36].

### GSPC based on a combination database

Previous data indicates that when bacteriophages and their bacterial hosts are included in genome signature-based phylogenies, bacteriophages tend to cluster together near their bacterial hosts [25]. We hypothesize that this clustering represents a limitation in the ability of bacteriophages to fully ameliorate to their host genome signature, and may be necessary for the bacteriophages to maintain host range [25]. The metagenomes from Bear Paw and Octopus hot springs, are limited to bacteriophages and archaeal viruses based on previous analysis of the contigs [12]. We constructed a database containing all sequenced Archaea, Bacteria, and viruses to determine if Bear Paw and Octopus metagenomic contigs have viral signatures or microbial signatures. Using GSPC based on this combination database, each of the contigs from Bear Paw and Octopus were classified similarly to their classification based on the viral database (Additional file 3), further suggesting their origin as bacteriophages and archaeal viruses.

### Other methods of metagenomic classification

Using Genbank BLAST algorithms, much of the Octopus metagenome has no identifiable homolog (Table 1). Homologs to only 23% of the Octopus contigs were identified, while 86% of the Bear Paw contigs had identifiable homologs (Table 1 and Figure 2, Panels B and D). Most of the homologs to Bear Paw contigs were from Bacteria, while Octopus contigs had many homologs to Archaea, Bacteria, and Eukarya. The lower proportion of identifiable homologs in Octopus compared to Bear Paw, suggests that an identification bias might exist based on a relative paucity of thermophilic viral sequences present in the Genbank database.

**Table 1:** Contig Identification Summary

	**Bear Paw**	**Octopus**
Number of Sequences	22	70

**Genbank**		

Archaea Hits	0 (0%)	9 (13%)
Extremophile^a ^Hits	4 (18%)	4 (6%)
Bacteria Hits	19 (86%)	7 (10%)
Eukaryote Hits	4 (18%)	9 (13%)

Total Identified	19 (86%)	16 (23%)

**Tetranucleotide GSPC**		

Archaea Hits	3 (14%)	56 (80%)
Extremophile^a ^Hits	6 (27%)	1 (1%)
Bacteria Hits	17 (77%)	13 (19%)

Total Identified	20 (91%)	69 (99%)

**Phylopythia**		

Archaea Hits	5 (23%)	26 (37%)
Extremophile^a ^Hits	0 (0%)	0 (0%)
Bacteria Hits	17 (77%)	26 (37%)
Eukaryote Hits	0 (0%)	12 (17%)

Total Identified	20 (91%)	64 (91%)

^a^Indicate hits to thermophilic bacteria

Another method for identification of metagenomic sequence fragments based on oligonucleotide sequence biases is the application Phylopythia, which uses a support vector machine to classify sequence fragments according to its database of oligonucleotide biases that includes Archaea, Bacteria, and Eukarya [20]. Previous data has demonstrated that for both bacterial and archaeal DNA fragments, the technique is quite robust in assigning fragments to different taxonomic classes [20]. While not developed specifically for bacteriophages, we applied Phylopythia towards the identification of metagenomic contigs from Bear Paw and Octopus. Phylopythia classified 91% of the sequence contigs from Bear Paw, and 91% from Octopus (Table 1). Many of the sequences could not be identified beyond the level of Kingdom or Class (Additional file 2). Some sequences were classified to eukaryotic Classes (including *Ascomycota*, *Insecta*, *Sordariomycetes*, and *Arthropoda*), bacterial Classes (including *Clostridia*, *Bacteroidetes*, *Gammaproteobacteria*, *Epsilonproteobacteria*, *Alphaproteobacteria*, and *Spirochaetes*), and archaeal Classes (including *Thermoprotei*, and *Methanomicrobia*) (Additional file 2). Since no Eukaryotes previously have been isolated at these temperatures [26], their viruses are unlikely to be members of these communities.

Using GSPC, Genbank BLAST, and Phylopythia, many of the metagenome contigs were classified to the archaeal Class *Thermoprotei*. Because of the apparent similarities in classifying contigs between each method, we used the Spearman non-parametric test to determine if there was a significant correlation between the classification predictions of each methodology. Because with certain methods, taxonomic classification was only possible at the level of Class (Additional file 2), we used the predicted Class of each contig for further evaluation. When examining the contigs, there was a high level of agreement between contig classification by GSPC based on a microbial database using either trinucleotides or tetranucleotides (Table 2). When comparing GSPC based on a microbial database using either criterion, both have significant agreement with those classification results of Genbank. There is no significant agreement between results of Genbank and Phylopythia, and less agreement between GSPC and Phylopythia. Nearly identical results were obtained using Kendall tau's non-parametric test (data not shown).

**Table 2:** Correlation between classification techniques by Class

			GSPC (Tetranucleotides)^a^	GSPC (Trinucleotides)^a^	Phylopythia	Genbank
Spearman's rho	GSPC (Tetranucleotides)	Correlation Coefficient	1.000	**0.650**	0.081	**0.422**
		Significance	NA	**0.000**	0.443	**0.000**
	GSPC (Trinucleotides)	Correlation Coefficient	**0.650**	1.000	0.225	**0.326**
		Significance	**0.000**	NA	0.032	**0.002**
	Phylopythia	Correlation Coefficient	0.081	0.225	1.000	0.086
		Significance	0.443	0.032	NA	0.417
	Genbank	Correlation Coefficient	**0.422**	**0.326**	0.086	1.000
		Significance	**0.000**	**0.002**	0.417	NA

Bold indicates correlation is significant at the < 0.01 level (2-tailed).

^a^Indicates GSPC based on a microbial database

## Discussion and conclusion

The exploration of microbial assemblages through microbiome genome analysis has provided insights into both community structure and physiology [1,2], but also has revealed a greater need for advances in technology to identify community constituents without significant homology in Genbank. Viral metagenomic analysis currently is substantially less well developed than that of cellular populations, and is limited by a low proportion of viral sequences compared to cellular sequences. Genome signature analysis is independent of nucleotide or amino acid alignments, and predicts relationships based on separate principles from those of BLAST search algorithms [15].

We sought to create two separate databases based on oligonucleotide frequencies of all sequenced microbial cellular and viral genomes, respectively, to determine whether known viruses could be used for accurate prediction of host microbe or virus ancestry. Our data demonstrate that when longer nucleotide sequences are available, GSPC makes more accurate predictions of both host and viral ancestry (Figures 1 and 8). As the length of nucleotide sequences decreases, GSPC accuracy also decreases (Figures 1 and 8). The heterogeneity of oligonucleotide signatures across certain bacteriophage genomes may explain why individual bacteriophage fragments are not always representative of their viral or host genomes [19,25].

Because previously sequenced viral metagenomes [13,40] are comprised mostly of single reads or smaller contigs, they generally are not amenable to GSPC analysis. Bear Paw and Octopus metagenomes are less diverse and have larger contigs [12], thus providing a more suitable dataset for GSPC. The GSPC method predicts many of the hot spring metagenomic contigs as archaeal viruses and thermophilic Bacteria (Figure 2), a finding that is consistent with the environment from which they were recovered. When homologs to the metagenomic contigs could be identified in Genbank, the presumptive hosts were generally consistent with the findings of GSPC (Additional file 2 and Table 2). While there was some agreement in contig prediction between GSPC and Phylopythia, many of the contigs were predicted to be derived from dissimilar organisms. Because GSPC predicts the origin of many of the contigs to be consistent with the known flora of these hot springs, while Phylopythia predicts many to have eukaryotic origin, we believe GSPC may provide a more specific methodology for contigs from such extreme environments.

We chose to analyze the metagenomes of two separate hot springs, Bear Paw and Octopus in Yellowstone National Park. Their conditions at the surface differ, suggesting there may be differences between the microbial flora present in each environment. Genbank BLAST and GSPC based on a microbial database both predict the origin of many Bear Paw contigs to have bacterial origin, while the viral database suggests the contigs are from bacteriophages (Additional file 3). In contrast, Genbank BLAST and GSPC based on a microbial database predict many Octopus contigs to have archaeal origin, with the viral database indicating many contigs may belong to archaeal virus Families *Fuselloviridae *and *Globuloviridae *(Additional file 3). In support of this finding, a previous metagenomic study of these hot springs detected homologs to nearly the entire genome of *Pyrobaculum *spherical virus [12], a member of the archaeal virus Family *Globuloviridae*. Although geochemistry has a large influence on the microbial composition of hot springs, microbial populations are highly temperature dependent [36]. We believe the bacterial predominance in Bear Paw hot spring compared to Octopus may be related to the lower temperature present in Bear Paw.

As greater numbers of viral communities are studied, new techniques for assessing metagenomic constituents are necessary. Previous studies of viral metagenomes have underscored the need for new techniques, as most of the available metagenomic sequences have limited detectable similarity to sequences in Genbank [9,11-13]. GSPC provides an approach complementary to BLAST search algorithms, taking advantage of properties of DNA patterns of nucleotide usage rather than nucleotide alignments. While not applicable to most lower temperature viral metagenomes due to the limited size of typical contigs in most studies, GSPC will likely become more suitable for analysis of these environments as next generation sequencing platforms allow collection of much larger amounts of sequence data and assembly of larger contigs. This will substantially increase the sensitivity in viral metagenomic studies in both predicting the host and classifying the types of viruses in the community. GSPC is a facile approach for classifying viral metagenomic sequences and inferring host relationships and is a highly complementary alternative to traditional BLAST searches, particularly when those searches fail to identify significant homology.

## Methods

### Virus collection and sequencing

Samples were collected in October 2003 from both Bear Paw and Octopus hot springs in the lower geyser basin in Yellowstone National Park. Viral particles were isolated, and libraries were constructed and sequenced and sequences were assembled as described [12]. Libraries from each hot spring were constructed using methods that select only for double stranded DNA viruses. We previously have based our minimum genome sequence length for analysis on the assumption that 95% of tetranucleotide combinations should occur at least 10 times [18,25]. The minimum genome length analyzed in this study was 1.9 kb (3.8 kb when analyzing both strands), which represents an assumption that 95% of tetranucleotide combinations should occur at least 7.5 times. Approximately 19.3% of the Bear Paw metagenomic contigs and 39.0% of the Octopus metagenomic contigs conformed to these criteria. Since hundreds or thousands of viral types inhabit Bear Paw and Octopus hot springs [12], these contigs represent only the most abundant viral types. Both metagenomes are available from the NCBI trace archive using CENTER_NAME = "JGI" and SEQ_LIB_ID = "AOIX" for Bear Paw sequences and SEQ_LIB_ID = "APNO" and SEQ_LIB_ID = "ATYB" for Octopus sequences.

### Oligonucleotide analysis

To determine oligonucleotide frequencies for genomes and metagenomic contigs, a Zero-Order Markov algorithm [41] was used, in which the expected number of oligonucleotides was determined by removing biases in mononucleotide frequencies, as determined by the equation: *E*(*W*) = [(A^a ^* C^c ^* G^g ^* T^t^) * N], where A, C, G, and T represent the frequency of the four nucleotides within the window being evaluated, respectively, a, c, g, and t represent the number of nucleotides A, C, G, and T in each oligonucleotide, respectively, and N represents the length of the genome or contig being evaluated [15]. The frequency of divergence for each oligonucleotide is expressed as the ratio of observed to expected, and were determined for each genome studied using Swaap Genome Search version 1.0.1 [42].

### Microbial and viral databases

A database was constructed containing all di-, tri-, tetra-, penta-, and hexanucleotide frequencies for all fully sequenced bacterial and archaeal genomes available in the Genbank database (383 genomes stored in the database on 5-21-07). Including separate chromosomes for certain organisms, there were 440 separate entries in the microbial database. A separate database was constructed for all di-, tri-, tetra-, penta-, and hexanucleotide frequencies for all known fully sequenced viruses using the Genbank database (3866 genomes stored in the database on 8-10-07).

### Genome signature-based phylogenetic classification

Genome signature-based phylogenetic classification (GSPC) was performed on individual metagenomic contigs, collective groups of metagenomic contigs, and viral fragments. Briefly, oligonucleotide frequencies were determined for all viral sequences, and Euclidean distances between each fragment and all frequencies in the databases were determined. Distances were determined by the equation: D_t _= 1/N^N ^* Σ|*F*_1_(*W*) - *F*_2_*(W)*|, where *F*_1_(*W*) and *F*_2_(*W*) represent *F*(*W*) for each of the oligonucleotides for any organisms or fragments 1 and 2, and N is the length of the oligonucleotide under evaluation [15,16]. Bootstrapping was performed by sampling with replacement of each of the oligonucleotide frequencies, phylograms were created using neighbor-joining analysis based on the resulting distance matrices using Swaap Genome Search 1.0.1 [42], reviewed via Paup 4.0b10 [43] or Treeview [44], and portions of phylogenies containing branches of interest were displayed using Corel Draw 11 (Corel Corp., Ottawa, Canada).

For the microbial database, contigs were classified based on their phylogenetic position, either monophyletic or paraphyletic. In cases where contigs were grouped monophyletically, they were classified based on the Kingdom, Phylum, Class, Order, Family, and Genus of that monophyletic group. When contigs were grouped paraphyletically, they were classified based on the Kingdom, Phylum, Class, Order, Family, and Genus of branches deep to that paraphyletic position. Example output of the sequence classification for the microbial database is demonstrated in Additional file 4. For the viral database, contigs were classified based on the DNA type, host type (bacterial or archaeal vs. eukaryal), viral type (Caudovirus vs. other), Family, and virus designation (e.g. T-7 like virus, etc...) based on the same principles as classification based on the microbial database.

### Analysis of known viruses

Oligonucleotide frequencies for known complete and partial viral genomes were determined using Swaap Genome Search version 1.0.1 [42]. A collection of 77 bacteriophages, for which hosts have been well described, were used for analysis of known viruses (Additional file 1). Each viral genome was assessed by GSPC using a microbial database, and results in accordance with their known hosts were determined. The percentage of viruses identified by Kingdom, Phylum, Class, Order, Family, and Genus of their known hosts were then determined.

For analysis of known viruses with the viral database, fragments rather than full-length viral genomes were used. Random bacteriophage and viral genomic fragments were generated because the viral database contains all known fully sequenced viruses, including the 77 bacteriophages used in our dataset. Random bacteriophage fragments of sizes 10,000 nucleotides, 5,000 nucleotides, and 2,000 nucleotides were generated using Swaap Genome Search 1.0.1 [42]. Five random fragments for each specified size were generated for each genome, and each was subjected to GSPC using a viral database. The percentage of viruses classified according to DNA type, virus type (bacteriophage or archaeal virus vs. eukaryotic virus), viral type (Caudovirus vs. other phage type), viral Family, and viral designation (e.g. T7-like viruses etc...) were then determined. The standard error was determined based on the compilation of 5 separate experiments.

### Other analysis of metagenomic contigs

All metagenomic contigs also were subjected to classification analysis using Phylopythia and Genbank tBLASTx analysis using the nonredundant database [14,20]. Hits were considered significant if the Expect values were less than 10^-3^.

Spearman's rho correlation test was performed on metagenome contigs using SPSS (SPSS Corp., Chicago, IL). Briefly, metagenome contigs were classified using Genbank, GSPC, or Phylopythia. The results of each method were compiled using the predicted Class of each contig, and each Class was coded using numbers 1 to 41. The resulting tables were then subjected to Spearman's rho correlation test or Kendall tau's correlation test using SPSS (SPSS Corp., Chicago, IL). Results were considered significant when p < 0.01.

## Abbreviations

GSPC: Genome Signature-based Phylogenetic Classification

## Authors' contributions

DP helped conceive of study, created databases, created GSPC, data analysis, manuscript preparation. TS helped conceive of study, metagenome preparation, data analysis, manuscript review.

## Supplementary Material

Additional file 1 **Phages used in this study.** Click here for file

Additional file 2 **Comparison of different classification methods.** Click here for file

Additional file 3 **Comparison of GSPC database classifications.** Click here for file

Additional file 4 **GSPC classification examples.** Click here for file
