# Supplementary material for: Genome signature analysis of thermal virus metagenomes reveals Archaea and thermophilic signatures
Source: BMC Genomics. 2008 Sep 17;9:420. doi: 10.1186/1471-2164-9-420 (PMC2556352; doi:10.1186/1471-2164-9-420)
Supplement: Additional file 1 — Phages used in this study. [file 1471-2164-9-420-S1.doc]

Supplemental Table 1

Summary of bacteriophages used in analysis

| **Phage** | **Size** | **Designation** | **Presumed Host Genus** | **Presumed Host Phylum** | **Presumed Host Class** | **Life Cycle** |
| --- | --- | --- | --- | --- | --- | --- |
| Phage 01205 | 43075 | Siphoviridae | Streptococcus | Firmicutes | Bacilli | Temperate |
| Phage 1 | 48178 | Myoviridae | Burkholderia | Proteobacteria | Betaproteobacteria | Temperate |
| Phage 1026b | 54865 | Siphoviridae | Burkholderia | Proteobacteria | Betaproteobacteria | Temperate |
| Phage 186 | 30624 | Myoviridae | Escherichia | Proteobacteria | Gammaproteobacteria | Temperate |
| Phage 22 | 63882 | Podoviridae | Burkholderia | Proteobacteria | Betaproteobacteria | Temperate |
| Phage 23 | 48023 | Myoviridae | Burkholderia | Proteobacteria | Betaproteobacteria | Temperate |
| Phage 2839 | 37618 | Siphoviridae | Listeria | Firmicutes | Bacilli | Temperate |
| Phage 77 | 41708 | Siphoviridae | Staphylococcus | Firmicutes | Cocci | Temperate |
| Phage 781 | 48248 | Myoviridae | Burkholderia | Proteobacteria | Betaproteobacteria | Temperate |
| Phage Barnyard | 70797 | Siphoviridae | Mycobacteria | Actinobacteria | Actinobacteridae | Temperate |
| Phage BIP-1 | 42638 | Podoviridae | Bordetella | Proteobacteria | Betaproteobacteria | Temperate |
| Phage BK5-T | 40003 | Siphoviridae | Lactococcus | Firmicutes | Bacilli | Temperate |
| Phage BMP-1 | 42663 | Podoviridae | Bordetella | Proteobacteria | Betaproteobacteria | Temperate |
| Phage BPP-1 | 42493 | Podoviridae | Bordetella | Proteobacteria | Betaproteobacteria | Temperate |
| Phage Bxz1 | 156102 | Myoviridae | Mycobacteria | Actinobacteria | Actinobacteridae | Temperate |
| Phage Bxz2 | 50913 | Siphoviridae | Mycobacteria | Actinobacteria | Actinobacteridae | Temperate |
| Phage Che8 | 59471 | Siphoviridae | Mycobacteria | Actinobacteria | Actinobacteridae | Temperate |
| Phage Che9c | 57050 | Siphoviridae | Mycobacteria | Actinobacteria | Actinobacteridae | Temperate |
| Phage Che9d | 56276 | Siphoviridae | Mycobacteria | Actinobacteria | Actinobacteridae | Temperate |
| Phage CJW1 | 75931 | Siphoviridae | Mycobacteria | Actinobacteria | Actinobacteridae | Temperate |
| Phage Corndog | 69777 | Siphoviridae | Mycobacteria | Actinobacteria | Actinobacteridae | Temperate |
| Phage D29 | 49136 | Siphoviridae | Mycobacteria | Actinobacteria | Actinobacteridae | Temperate |
| Phage D3 | 56425 | Siphoviridae | Pseudomonas | Proteobacteria | Gammaproteobacteria | Temperate |
| Phage D3112 | 37611 | Siphoviridae | Pseudomonas | Proteobacteria | Gammaproteobacteria | Temperate |
| Phage DT1 | 34815 | Siphoviridae | Streptococcus | Firmicutes | Bacilli | Temperate |
| Phage EJ1 | 42935 | Myoviridae | Streptococcus | Firmicutes | Bacilli | Temperate |
| Phage Epsilon 15 | 39671 | Podoviridae | Salmonella | Proteobacteria | Gammaproteobacteria | Temperate |
| Phage GA-1 | 21129 | Podoviridae | Bacillus | Firmicutes | Bacilli | Lytic |
| Phage GH1 | 37359 | Podoviridae | Pseudomonas | Proteobacteria | Gammaproteobacteria | Temperate |
| Phage HK022 | 40751 | Siphoviridae | Escherichia | Proteobacteria | Gammaproteobacteria | Temperate |
| Phage HP1 | 32355 | Myoviridae | Haemophilus | Proteobacteria | Gammaproteobacteria | Temperate |
| Phage HP2 | 31508 | Myoviridae | Haemophilus | Proteobacteria | Gammaproteobacteria | Temperate |
| Phage J928 | 38384 | Siphoviridae | Lactobacillus | Firmicutes | Bacilli | Temperate |
| Phage K139 | 33106 | Myoviridae | Vibrio | Proteobacteria | Gammaproteobacteria | Temperate |
| Phage L5 | 52297 | Siphoviridae | Mycobacteria | Actinobacteria | Actinobacteridae | Temperate |
| Phage Lambda | 48502 | Siphoviridae | Escherichia | Proteobacteria | Gammaproteobacteria | Temperate |
| Phage LJ965 | 40190 | Siphoviridae | Lactobacillus | Firmicutes | Bacilli | Temperate |
| Phage MM1 | 42935 | Myoviridae | Streptococcus | Firmicutes | Bacilli | Temperate |
| Phage Mu | 36717 | Myoviridae | Escherichia | Proteobacteria | Gammaproteobacteria | Temperate |
| Phage Omega | 110865 | Siphoviridae | Mycobacteria | Actinobacteria | Actinobacteridae | Temperate |
| Phage P22 | 41724 | Podoviridae | Salmonella | Proteobacteria | Gammaproteobacteria | Temperate |
| Phage P335 | 36596 | Siphoviridae | Lactococcus | Firmicutes | Bacilli | Temperate |
| Phage P4 | 11624 | Myoviridae | Escherichia | Proteobacteria | Gammaproteobacteria | Temperate |
| Phage PG1 | 68999 | Siphoviridae | Mycobacteria | Actinobacteria | Actinobacteridae | Temperate |
| Phage Phi 105 | 39325 | Siphoviridae | Bacillus | Firmicutes | Bacilli | Temperate |
| Phage Phi 11 | 43604 | Siphoviridae | Staphylococcus | Firmicutes | Cocci | Temperate |
| Phage Phi 12 | 44970 | Siphoviridae | Staphylococcus | Firmicutes | Cocci | Temperate |
| Phage Phi 13 | 42722 | Siphoviridae | Staphylococcus | Firmicutes | Cocci | Temperate |
| Phage Phi 4795 | 57930 | Siphoviridae | Escherichia | Proteobacteria | Gammaproteobacteria | Temperate |
| Phage Phi A1122 | 37555 | Podoviridae | Yersinia | Proteobacteria | Gammaproteobacteria | Lytic |
| Phage Phi ADH | 43785 | Siphoviridae | Lactobacillus | Firmicutes | Bacilli | Temperate |
| Phage Phi BT1 | 41831 | Siphoviridae | Streptomyces | Actinobacteria | Actinobacteridae | Temperate |
| Phage Phi C31 | 41491 | Siphoviridae | Streptomyces | Actinobacteria | Actinobacteridae | Temperate |
| Phage Phi CTX | 35559 | Myoviridae | Pseudomonas | Proteobacteria | Gammaproteobacteria | Temperate |
| Phage Phi KMV | 42519 | Podoviridae | Pseudomonas | Proteobacteria | Gammaproteobacteria | Lytic |
| Phage Phi KO2 | 51601 | Siphoviridae | Klebsiella | Proteobacteria | Gammaproteobacteria | Temperate |
| Phage Phi LC3 | 32172 | Siphoviridae | Lactococcus | Firmicutes | Bacilli | Temperate |
| Phage Phi PV83 | 45636 | Siphoviridae | Staphylococcus | Firmicutes | Cocci | Temperate |
| Phage Phi SLT | 42942 | Siphoviridae | Staphylococcus | Firmicutes | Cocci | Temperate |
| Phage PVL | 41401 | Siphoviridae | Staphylococcus | Firmicutes | Cocci | Temperate |
| Phage PZA | 19366 | Podoviridae | Bacillus | Firmicutes | Bacilli | Lytic |
| Phage RB49 | 164018 | Myoviridae | Escherichia | Proteobacteria | Gammaproteobacteria | Temperate |
| Phage Rosebush | 67480 | Siphoviridae | Mycobacteria | Actinobacteria | Actinobacteridae | Temperate |
| Phage SF6 | 39043 | Podoviridae | Shigella | Proteobacteria | Gammaproteobacteria | Temperate |
| Phage SP6 | 43769 | Podoviridae | Salmonella | Proteobacteria | Gammaproteobacteria | Lytic |
| Phage SPP1 | 44007 | Siphoviridae | Bacillus | Firmicutes | Bacilli | Temperate |
| Phage ST64T | 40679 | Podoviridae | Salmonella | Proteobacteria | Gammaproteobacteria | Temperate |
| Phage Stx1 | 59866 | Siphoviridae | Escherichia | Proteobacteria | Gammaproteobacteria | Temperate |
| Phage Stx2 | 62706 | Siphoviridae | Escherichia | Proteobacteria | Gammaproteobacteria | Temperate |
| Phage T1 | 48836 | Siphoviridae | Escherichia | Proteobacteria | Gammaproteobacteria | Temperate |
| Phage T3 | 38208 | Podoviridae | Escherichia | Proteobacteria | Gammaproteobacteria | Lytic |
| Phage T7 | 39937 | Podoviridae | Escherichia | Proteobacteria | Gammaproteobacteria | Lytic |
| Phage U136 | 36798 | Siphoviridae | Lactococcus | Firmicutes | Bacilli | Temperate |
| Phage V | 37074 | Podoviridae | Shigella | Proteobacteria | Gammaproteobacteria | Temperate |
| Phage VWB | 49220 | Siphoviridae | Streptomyces | Actinobacteria | Actinobacteridae | Temperate |
| Phage YEO3-12 | 39600 | Podoviridae | Yersenia | Proteobacteria | Gammaproteobacteria | Lytic |
