# Supplementary material for: Genome signature analysis of thermal virus metagenomes reveals Archaea and thermophilic signatures
Source: BMC Genomics. 2008 Sep 17;9:420. doi: 10.1186/1471-2164-9-420 (PMC2556352; doi:10.1186/1471-2164-9-420)
Supplement: Additional file 2 — Comparison of different classification methods. [file 1471-2164-9-420-S2.doc]

Supplemental Table 2

Comparison of classification methods

| **Contig** | **Tetranucleotide GSPC^a,b^** | **Phylopythia^b^** | **Genbank tblastx^b^** |
| --- | --- | --- | --- |
| Octopus_549 | Pyrobaculum (57%)^c^ | Bacteria | Clostridium |
| Octopus_8149 | Mycobacterium (20%) | Insecta | Mus musculus |
| Octopus_1636 | Bacteria (78%) | No Classification | Bacteriophage BFK20 |
| Octopus_7654 | Pyrobaculum (94%) | Crenarchaeota | Thermus |
| Octopus_3453 | Pyrobaculum (69%) | Crenarchaeota | Sulfolobus |
| Octopus_9974 | Aeropyrum (96%) | Euryarchaeota | Sulfolobus |
| Octopus_6453 | Pyrobaculum (76%) | Bacteria | Pyrobaculum |
| Octopus_2607 | Pyrobaculum (59%) | No Classification | Clostridium |
| Octopus_4946 | Pyrobaculum (15%) | Ascomycota | No Classification |
| Octopus_3251 | Pyrobaculum (59%) | Euryarchaeota | No Classification |
| Octopus_2468 | Aeropyrum (99%) | Thermoprotei | Mus musculus |
| Octopus_773 | Pyrobaculum (38%) | Clostridia | No Classification |
| Octopus_345 | Pyrobaculum (41%) | Bacteria | Mus musculus |
| Octopus_369 | Pyrobaculum (41%) | Sordariomycetes | Acidianu |
| Octopus_4639 | Archaea (74%) | Euryarchaeota | Acidianus |
| Octopus_1497 | Pyrobaculum (86%) | Thermoprotei | No Classification |
| Octopus_179 | Pyrobaculum (51%) | Bacteria | No Classification |
| Octopus_1248 | Pyrobaculum (57%) | Bacteroidetes | No Classification |
| Octopus_8849 | Pyrobaculum (57%) | Bacteroidetes | Zebrafish |
| Octopus_218 | Treponema (83%) | Bacteria | No Classification |
| Octopus_5028 | Pyrobaculum (60%) | Insecta | No Classification |
| Octopus_2598 | Pyrobaculum (63%) | Bacteria | Methanosarcina |
| Octopus_3107 | Pyrobaculum (63%) | Bacteria | No Classification |
| Octopus_5846 | Pyrobaculum (48%) | Ascomycota | Acidianus |
| Octopus_8626 | Pyrobaculum (39%) | Ascomycota | Dechloromonas |
| Octopus_352 | Pyrobaculum (62%) | Archaea | Pyrobaculum |
| Octopus_4 | Pyrobaculum (91%) | No Classification | Homo sapiens |
| Octopus_6297 | Pyrobaculum (74%) | Archaea | Spiromyces |
| Octopus_10390 | Aeropyrum (77%) | Thermoprotei | Acidianus |
| Octopus_2222 | Pyrobaculum (88%) | Thermoprotei | Sulfolobus |
| Octopus_322 | Pyrobaculum (42%) | Archaea | Acidianus |
| Octopus_4475 | Mycobacterium (19%) | Archaea | Acidianus |
| Octopus_6378 | Anaplasmataceae (1%) | Ascomycota | Methanosarcina |
| Octopus_138 | Pyrobaculum (20%) | Arthropoda | Campylocentrum |
| Octopus_90 | Mycobacterium (56%) | Archaea | No Classification |
| Octopus_6791 | Thermoplasma (11%) | Methanomicrobia | No Classification |
| Octopus_1303 | Pyrobaculum (52%) | Gammaproteobacteria | Oryza |
| Octopus_552 | Pyrobaculum (91%) | Insecta | No Classification |
| Octopus_1468 | Pyrobaculum (99%) | Bacteria | Homo Sapiens |
| Octopus_3103 | Pyrobaculum (36%) | Euryarchaeota | No Classification |
| Octopus_48 | Pyrobaculum (67%) | Archaea | No Classification |
| Octopus_6841 | Pyrobaculum (42%) | Archaea | Sulfolobus |
| Octopus_2338 | Pyrobaculum (32%) | Gammaproteobacteria | Methanosarcina |
| Octopus_2913 | Mycobacterium (48%) | Archaea | Pyrobaculum |
| Octopus_10681 | Aeropyrum (92%) | Euryarchaeota | No Classification |
| Octopus_1255 | Pyrobaculum (68%) | Archaea | No Classification |
| Octopus_1691 | Pyrobaculum (21%) | Proteobacteria | Pyrobaculum |
| Octopus_1462 | Pyrobaculum (42%) | Bacteria | Staphylococcus phage |
| Octopus_1653 | Pyrobaculum (46%) | Gammaproteobacteria | Sulfolobus |
| Octopus_303 | Proteobacteria (6%) | Archaea | Agrobacterium |
| Octopus_3470 | Pyrobaculum (46%) | Bacteria | No Classification |
| Octopus_9263 | Thermus (93%) | Archaea | Aquifex |
| Octopus_6151 | Pyrobaculum (47%) | Arthropoda | Bacillus |
| Octopus_8212 | Pyrobaculum (37%) | Bacteria | No Classification |
| Octopus_163 | Pyrobaculum (43%) | Insecta | No Classification |
| Octopus_393 | Pyrobaculum (19%) | Bacteria | No Classification |
| Octopus_6866 | Bacteria (71%) | Bacteria | No Classification |
| Octopus_2025 | Pyrobaculum (38%) | Bacteria | Homo Sapiens |
| Octopus_275 | Pyrobaculum (29%) | Bacteria | No Classification |
| Octopus_3503 | Aeropyrum (97%) | Thermoprotei | Aeropyrum |
| Octopus_2946 | Aeropyrum (100%) | Thermoprotei | Aeropyrum |
| Octopus_586 | Tropheryma (1%) | Bacteroidetes | Geobacter |
| Octopus_183 | Pyrobaculum (18%) | Arthropoda | No Classification |
| Octopus_2448 | Pyrobaculum (52%) | Bacteroidetes | No Classification |
| Octopus_4401 | Pyrobaculum (37%) | Bacteroidetes | No Classification |
| Octopus_157 | Pyrobaculum (98%) | Insecta | No Classification |
| Octopus_113 | Bacteria (76%) | No Classification | No Classification |
| Octopus_7030 | Treponema (68%) | Bacteria | No Classification |
| Octopus_5501 | Pyrobaculum (68%) | Euryarchaeota | Acidianus |
| Bearpaw_697 | Bacteroides (17%) | Bacteroidetes | Trypanosoma |
| Bearpaw_1913 | Zymomonas (82%) | Alphaproteobacteria | Chromobacterium |
| Bearpaw_3850 | Leptospira (100%) | Spirochaetes | Porphyromonas |
| Bearpaw_1141 | Proteobacteria (47%) | Bacteria | Homo sapiens |
| Bearpaw_252 | Bacteria (98%) | Epsilonproteobacteria | Mus musculus |
| Bearpaw_2800 | Syntrophus (78%) | Methanomicrobia | Unidentified phage |
| Bearpaw_2107 | Leptospira (63%) | Spirochaetes | Chlorobium |
| Bearpaw_4421 | No Classification (0%) | Bacteroidetes | Bacteroidetes |
| Bearpaw_1453 | Leptospira (43%) | Bacteroidetes | Bacteroidetes |
| Bearpaw_339 | Methanococcus (25%) | Bacteroidetes | Flavobacterium |
| Bearpaw_3034 | Thermus (79%) | Archaea | Aquifex |
| Bearpaw_2031 | Thermus (63%) | Archaea | Aquifex |
| Bearpaw_3730 | Leptospira (59%) | Spirochaetes | Legionella |
| Bearpaw_4212 | Leptospira (71%) | Spirochaetes | Streptococcus |
| Bearpaw_2081 | Pyrobaculum (53%) | Euryarchaeota | Acidianus |
| Bearpaw_2142 | Nanoarchaeum (25%) | Archaea | Aquifex |
| Bearpaw_2037 | Synechococcus (45%) | Gammaproteobacteria | Photobacterium |
| Bearpaw_24 | Thermus (48%) | Epsilonproteobacteria | Aquifex |
| Bearpaw_169 | Chlamydophila (26%) | Bacteroidetes | Flavobacterium |
| Bearpaw_616 | Pyrobaculum (33%) | Bacteria | Sphingomonas |
| Bearpaw_205 | Proteobacteria (29%) | Gammaproteobacteria | Burkholderia |
| Bearpaw_1538 | Lactobacillus (49%) | Bacteroidetes | Plasmodium |

^a^Indicates Tetranucleotide GSPC based on a microbial database.

^b^Classification by Genus when available, however, methods may only be able to classify contigs by

Kingdom or Class

^c^Number in parenthesis indicates percentage occurred based on 100 bootstrap replicates
