# Supplementary material for: Genome signature analysis of thermal virus metagenomes reveals Archaea and thermophilic signatures
Source: BMC Genomics. 2008 Sep 17;9:420. doi: 10.1186/1471-2164-9-420 (PMC2556352; doi:10.1186/1471-2164-9-420)
Supplement: Additional file 3 — Comparison of GSPC database classifications. [file 1471-2164-9-420-S3.doc]

Supplemental Table 3

Comparison of database classifications

| **Contig** | **Size** | **Microbial Database^a^** | **Viral database^b^** | **Combined Database^c^** |
| --- | --- | --- | --- | --- |
| Octopus_549 | 4551 | Archaea – Thermoprotei | Bacteriophage - Caudoviridae | Bacteriophage - Caudoviridae |
| Octopus_8149 | 4517 | Bacteria – Actinobacteridae | No Classification | No Classification |
| Octopus_1636 | 4495 | Bacteria | RNA virus | RNA virus |
| Octopus_7654 | 4019 | Archaea – Thermoprotei | Archaeal virus - Globuloviridae | Archaeal virus - Globuloviridae |
| Octopus_3453 | 3890 | Archaea – Thermoprotei | Archaeal virus - Globuloviridae | Archaeal virus - Globuloviridae |
| Octopus_9974 | 3836 | Archaea – Thermoprotei | Bacteriophage - Podoviridae | Bacteriophage - Podoviridae |
| Octopus_6453 | 3811 | Archaea – Thermoprotei | Archaeal virus - Globuloviridae | Archaeal virus - Globuloviridae |
| Octopus_2607 | 3618 | Archaea – Thermoprotei | Bacteriophage - Siphoviridae | Bacteriophage - Siphoviridae |
| Octopus_4946 | 3480 | Archaea – Thermoprotei | Bacteriophage - Other | Bacteriophage - Other |
| Octopus_3251 | 3463 | Archaea – Thermoprotei | Bacteriophage - Other | Bacteriophage - Other |
| Octopus_2468 | 3374 | Archaea – Thermoprotei | Bacteriophage - Myoviridae | Bacteriophage - Myoviridae |
| Octopus_773 | 3321 | Archaea – Thermoprotei | Bacteriophage - Other | Bacteriophage - Other |
| Octopus_345 | 3257 | Archaea – Thermoprotei | No Classification | No Classification |
| Octopus_369 | 3206 | Archaea – Thermoprotei | Bacteriophage - Other | Bacteriophage - Other |
| Octopus_4639 | 3187 | Archaea | Bacteriophage - Other | Bacteriophage - Other |
| Octopus_1497 | 3164 | Archaea – Thermoprotei | Archaeal virus - Globuloviridae | Archaeal virus - Globuloviridae |
| Octopus_179 | 3133 | Archaea – Thermoprotei | Bacteriophage - Other | Bacteriophage - Other |
| Octopus_1248 | 3066 | Archaea – Thermoprotei | No Classification | No Classification |
| Octopus_8849 | 3065 | Archaea – Thermoprotei | Archaeal virus - Globuloviridae | Archaeal virus - Globuloviridae |
| Octopus_218 | 3037 | Bacteria – Spirochaetes | Bacteriophage - Caudoviridae | Bacteriophage - Caudoviridae |
| Octopus_5028 | 2957 | Archaea – Thermoprotei | Bacteriophage - Other | Bacteriophage - Other |
| Octopus_2598 | 2948 | Archaea – Thermoprotei | Archaeal virus - Fuselloviridae | Archaeal virus - Fuselloviridae |
| Octopus_3107 | 2941 | Archaea – Thermoprotei | Bacteriophage - Podoviridae | Bacteriophage - Podoviridae |
| Octopus_5846 | 2893 | Archaea – Thermoprotei | Bacteriophage - Other | Bacteriophage - Other |
| Octopus_8626 | 2893 | Archaea – Thermoprotei | No Classification | No Classification |
| Octopus_352 | 2871 | Archaea – Thermoprotei | Archaeal virus - Globuloviridae | Archaeal virus - Globuloviridae |
| Octopus_4 | 2655 | Archaea – Thermoprotei | Archaeal virus - Globuloviridae | Archaeal virus - Globuloviridae |
| Octopus_6297 | 2797 | Archaea – Thermoprotei | No Classification | No Classification |
| Octopus_10390 | 2805 | Archaea – Thermoprotei | Bacteriophage - Other | Bacteriophage - Other |
| Octopus_2222 | 2778 | Archaea – Thermoprotei | Archaeal virus - Globuloviridae | Archaeal virus - Globuloviridae |
| Octopus_322 | 2740 | Archaea – Thermoprotei | RNA virus | RNA virus |
| Octopus_4475 | 2734 | Bacteria – Actinobacteridae | RNA virus | RNA virus |
| Octopus_6378 | 2683 | Bacteria – Alphaproteobacteria | Bacteriophage - Other | Bacteriophage - Other |
| Octopus_138 | 2690 | Archaea – Thermoprotei | Archaeal virus - Globuloviridae | Archaeal virus - Globuloviridae |
| Octopus_90 | 2677 | Bacteria – Actinobacteridae | RNA virus | RNA virus |
| Octopus_6791 | 2686 | Archaea – Thermoplasmata | No Classification | No Classification |
| Octopus_1303 | 2676 | Archaea – Thermoprotei | Archaeal virus - Globuloviridae | Archaeal virus - Globuloviridae |
| Octopus_552 | 2679 | Archaea – Thermoprotei | Archaeal virus - Globuloviridae | Archaeal virus - Globuloviridae |
| Octopus_1468 | 2670 | Archaea – Thermoprotei | Archaeal virus - Globuloviridae | Archaeal virus - Globuloviridae |
| Octopus_3103 | 2670 | Archaea – Thermoprotei | No Classification | No Classification |
| Octopus_48 | 2659 | Archaea – Thermoprotei | Archaeal virus - Globuloviridae | Archaeal virus - Globuloviridae |
| Octopus_6841 | 2653 | Archaea – Thermoprotei | Bacteriophage - Caudoviridae | Bacteriophage - Caudoviridae |
| Octopus_2338 | 2608 | Archaea – Thermoprotei | No Classification | No Classification |
| Octopus_2913 | 2610 | Bacteria – Actinobacteridae | No Classification | No Classification |
| Octopus_10681 | 2608 | Archaea – Thermoprotei | No Classification | No Classification |
| Octopus_1255 | 2588 | Archaea – Thermoprotei | Archaeal virus - Globuloviridae | Archaeal virus - Globuloviridae |
| Octopus_1691 | 2583 | Archaea – Thermoprotei | Bacteriophage - Caudoviridae | Bacteriophage - Caudoviridae |
| Octopus_1462 | 2570 | Archaea – Thermoprotei | No Classification | No Classification |
| Octopus_1653 | 2581 | Archaea – Thermoprotei | Archaeal virus - Fuselloviridae | Archaeal virus - Fuselloviridae |
| Octopus_303 | 2571 | Bacteria – Proteobacteria | Bacteriophage - Myoviridae | Bacteriophage - Myoviridae |
| Octopus_3470 | 2571 | Archaea – Thermoprotei | Bacteriophage - Other | Bacteriophage - Other |
| Octopus_9263 | 2571 | Bacteria – Deinococci | Bacteriophage - Podoviridae | Bacteriophage - Podoviridae |
| Octopus_6151 | 2564 | Archaea – Thermoprotei | Bacteriophage - Caudoviridae | Bacteriophage - Caudoviridae |
| Octopus_8212 | 2564 | Archaea – Thermoprotei | Bacteriophage - Other | Bacteriophage - Other |
| Octopus_163 | 2530 | Archaea – Thermoprotei | No Classification | No Classification |
| Octopus_393 | 2558 | Archaea – Thermoprotei | Bacteriophage - Other | Bacteriophage - Other |
| Octopus_6866 | 2558 | Bacteria | RNA virus | RNA virus |
| Octopus_2025 | 2552 | Archaea – Thermoprotei | Bacteriophage - Other | Bacteriophage - Other |
| Octopus_275 | 2534 | Archaea – Thermoprotei | Bacteriophage - Other | Bacteriophage - Other |
| Octopus_3503 | 2535 | Archaea – Thermoprotei | Bacteriophage - Myoviridae | Bacteriophage - Myoviridae |
| Octopus_2946 | 2538 | Archaea – Thermoprotei | Bacteriophage - Myoviridae | Bacteriophage - Myoviridae |
| Octopus_586 | 2530 | Bacteria – Actinobacteridae | Bacteriophage - Caudoviridae | Bacteriophage - Caudoviridae |
| Octopus_183 | 2489 | Archaea – Thermoprotei | RNA virus | RNA virus |
| Octopus_2448 | 2481 | Archaea – Thermoprotei | Bacteriophage - Other | Bacteriophage - Other |
| Octopus_4401 | 2498 | Archaea – Thermoprotei | Bacteriophage - Siphoviridae | Bacteriophage - Siphoviridae |
| Octopus_157 | 2466 | Archaea – Thermoprotei | Archaeal virus - Globuloviridae | Archaeal virus - Globuloviridae |
| Octopus_113 | 2455 | Bacteria | Bacteriophage - Other | Bacteriophage - Other |
| Octopus_7030 | 2471 | Bacteria – Spirochaetes | Bacteriophage - Siphoviridae | Bacteriophage - Siphoviridae |
| Octopus_5501 | 2463 | Archaea – Thermoprotei | RNA virus | RNA virus |
| Bearpaw_697 | 3271 | Bacteria – Bacteroidetes | Bacteriophage - Myoviridae | Bacteriophage - Myoviridae |
| Bearpaw_1913 | 2734 | Bacteria – Alphaproteobacteria | No Classification | No Classification |
| Bearpaw_3850 | 2709 | Bacteria – Spirochaetes | Bacteriophage - Myoviridae | Bacteriophage - Myoviridae |
| Bearpaw_1141 | 2517 | Bacteria – Proteobacteria | Bacteriophage - Other | Bacteriophage - Other |
| Bearpaw_252 | 2487 | Bacteria | Bacteriophage - Other | Bacteriophage - Other |
| Bearpaw_2800 | 2302 | Bacteria – Deltaproteobacteria | Bacteriophage - Myoviridae | Bacteriophage - Myoviridae |
| Bearpaw_2107 | 2247 | Bacteria – Spirochaetes | Bacteriophage - Myoviridae | Bacteriophage - Myoviridae |
| Bearpaw_4421 | 2221 | No Classification | Bacteriophage - Other | Bacteriophage - Other |
| Bearpaw_1453 | 2166 | Bacteria – Spirochaetes | Bacteriophage - Myoviridae | Bacteriophage - Myoviridae |
| Bearpaw_339 | 1866 | Archaea – Methanococci | Bacteriophage - Myoviridae | Bacteriophage - Myoviridae |
| Bearpaw_3034 | 2172 | Bacteria – Deinococci | No Classification | No Classification |
| Bearpaw_2031 | 2137 | Bacteria – Deinococci | Bacteriophage - Other | Bacteriophage - Other |
| Bearpaw_3730 | 2150 | Bacteria – Spirochaetes | Bacteriophage - Other | Bacteriophage - Other |
| Bearpaw_4212 | 2101 | Bacteria – Spirochaetes | Bacteriophage - Myoviridae | Bacteriophage - Myoviridae |
| Bearpaw_2081 | 2099 | Archaea – Thermoprotei | Bacteriophage - Other | Bacteriophage - Other |
| Bearpaw_2142 | 2075 | Archaea – Nanoarchaeota | No Classification | No Classification |
| Bearpaw_2037 | 2069 | Bacteria – Cyanobacteria | Bacteriophage - Other | Bacteriophage - Other |
| Bearpaw_24 | 2053 | Bacteria – Deinococci | Bacteriophage - Podoviridae | Bacteriophage - Podoviridae |
| Bearpaw_169 | 2051 | Bacteria – Chlamydiae | Bacteriophage - Other | Bacteriophage - Other |
| Bearpaw_616 | 1968 | Archaea – Thermoprotei | Bacteriophage - Other | Bacteriophage - Other |
| Bearpaw_205 | 2039 | Bacteria – Proteobacteria | Bacteriophage - Other | Bacteriophage - Other |
| Bearpaw_1538 | 1993 | Bacteria – Bacilli | Bacteriophage - Siphoviridae | Bacteriophage - Siphoviridae |

^a^Indicates Tetranucleotide GSPC based on a microbial database by Class

^b^Indicates Tetranucleotide GSPC based on a viral database by Family

^c^Indicates Tetranucleotide GSPC based on a combined microbial and viral database by Family
