# Supplementary material for: Genome signature analysis of thermal virus metagenomes reveals Archaea and thermophilic signatures
Source: BMC Genomics. 2008 Sep 17;9:420. doi: 10.1186/1471-2164-9-420 (PMC2556352; doi:10.1186/1471-2164-9-420)
Supplement: Additional file 4 — GSPC classification examples. [file 1471-2164-9-420-S4.doc]

**Supplemental Table 4**

**Examples of Contig** Classification Process

| **Bear Paw 24** | | | |
| --- | --- | --- | --- |
|  | **Monophyletic Groups** | **Percent Occurred** | **Number Occurred** |
|  | No Groups | 68.00 | 68.00 |
|  | Nanoarchaeum_equitans | 30.00 | 30.00 |
|  | Aquifex_aeolicus | 2.00 | 2.00 |
|  |  |  |  |
|  | **Classification** |  |  |
|  | Bacteria | 68.75 | 68.80 |
|  | Deinococcus-Thermus | 47.50 | 47.50 |
|  | Deinococci | 47.50 | 47.50 |
|  | Thermales | 47.50 | 47.50 |
|  | Thermaceae | 47.50 | 47.50 |
|  | Thermus | 47.50 | 47.50 |
|  | Archaea | 30.00 | 30.00 |
|  | Nanoarchaeota | 30.00 | 30.00 |
|  | Nanoarchaeum | 30.00 | 30.00 |
|  | Aquificae | 2.00 | 2.00 |
|  | Aquificales | 2.00 | 2.00 |
|  | Aquificaceae | 2.00 | 2.00 |
|  | Aquifex | 2.00 | 2.00 |
|  |  |  |  |
| **Octopus 9974** | | | |
|  | **Monophyletic With** | **Percent Occurred** | **Number Occurred** |
|  | Aeropyrum_pernix | 96.00 | 96.00 |
|  | No Groups | 4.00 | 4.00 |
|  |  |  |  |
|  | **Classification** |  |  |
|  | Archaea | 98.00 | 98.00 |
|  | Crenarchaeota | 96.00 | 96.00 |
|  | Thermoprotei | 96.00 | 96.00 |
|  | Desulfurococcales | 96.00 | 96.00 |
|  | Desulfurococcaceae | 96.00 | 96.00 |
|  | Aeropyrum | 96.00 | 96.00 |
|  | Euryarchaeota | 1.10 | 1.10 |
|  | Thermococci | 1.10 | 1.10 |
|  | Thermococcales | 1.10 | 1.10 |
|  | Thermococcaceae | 1.10 | 1.10 |
|  | Pyrococcus | 1.10 | 1.10 |
